# Prognostic value of programmed cell death ligand 1 expression in patients with head and neck cancer: A systematic review and meta-analysis

**DOI:** 10.1371/journal.pone.0179536

**Published:** 2017-06-12

**Authors:** Ji Li, Ping Wang, Youliang Xu

**Affiliations:** 1 Department of Stomatology, Huanggang Central Hospital, Huanggang, Hubei, China; 2 Department of Oncology, Huanggang Central Hospital, Huanggang, Hubei, China; 3 Department of Stomatology, The People's Hospital of Tuanfeng, Huanggang, Hubei, China; Seoul National University College of Pharmacy, REPUBLIC OF KOREA

## Abstract

**Background:**

Programmed cell death ligand 1 (PD-L1) expression was reported to be correlated with poor prognosis in various cancers. However, the relationship between PD-L1 expression and the survival of patients with head and neck cancer (HNC) remains inconclusive. In the present study, we aimed to clarify the prognostic value of PD-L1 in HNC patients using meta-analysis techniques.

**Methods:**

A comprehensive database searching was conducted in the PubMed, EMBASE, Web of Science and Cochrane Library from inception to August 2016. Studies meeting the inclusion criteria were included. The methodological quality of included studies was assessed by the Newcastle-Ottawa quality assessment scale. Hazard ratios (HRs) with their corresponding 95% confidence intervals (CIs) were pooled by STATA 11.0 for the outcome of overall survival (OS) and disease-free survival (DFS).

**Results:**

A total of 17 studies with 2,869 HNC patients were included in the meta-analysis. The results of meta-analysis showed that there was no significant correlation between PD-L1 expression and OS (HR, 1.23; 95% CI, 0.99–1.53; *P* = 0.065) or DFS (HR, 1.42; 95% CI, 1.00–2.03; *P* = 0.052) of HNC patients. However, the subgroup analysis suggested that positive expression of PD-L1 was associated with poor OS (HR, 1.38; 95% CI, 1.12, 1.70; *P* = 0.003) and DFS (HR, 1.99; 95% CI, 1.59, 2.48; *P* = 0.001) in HNC patients from Asian countries/regions. The subgroup analysis also showed that the correlations between PD-L1 and prognosis are variant among different subtypes of HNC. When performing sensitive analyses, we found that the results of meta-analyses were not robust.

**Conclusion:**

The meta-analysis indicated that positive expression of PD-L1 could serve as a good predictor for poor prognosis of Asian patients with HNC. However, the findings still need to be confirmed by large-scale, prospective studies.

## Introduction

Head and neck cancer (HNC) is a group of cancers that starts within the oral cavity, nasal cavity, larynx, pharynx, or salivary glands [1]. According to new data from the Global Burden of Disease Study 2013, HNC globally affected more than 4.6 million people and resulted in more than 362,000 deaths [2, 3], which made HNC the seventh most frequent cancer and the ninth most frequent cause of death from cancer. Despite the improvement of surgical techniques, as well as the introduction of radiotherapy and chemotherapy, the survival rate of HNC patients was still not significantly improved in decades [4, 5]. Therefore, it is urgent to find new therapeutic targets, which needs to uncover the biological mechanisms underlying the carcinogenesis of HNC and discover critical biomarkers for predicting prognosis of HNC.

It is well known that endogenous immune responses could recognize and eradicate malignant cells, and the ability of cancer cells to evade immune surveillance has been recognized as a distinct hallmark of cancer [6, 7]. Recently, programmed cell death ligand 1 (PD-L1) was demonstrated to be involved in the immune escape mechanism of cancer cells [8, 9]. PD-L1 is a surface glycoprotein that belongs to the B7/CD28 co-stimulatory factor superfamily [10]. In normal tissues, PD-L1 is limitedly expressed and interacts with its receptor, programmed cell death 1 (PD-1), to protect healthy cells from excessive inflammatory or autoimmune responses [11, 12]. However, interactions between PD-L1 and PD-1 in the tumor microenvironment could inhibit the proliferation of activated T-cells and promote the apoptosis of T-cells, resulting in enhanced tumor cell growth [6, 13]. Positive expression of PD-L1 was observed in various malignancies, and has been suggested to be a negative prognostic factor in breast cancer [14], renal cell cancer [15], and gastrointestinal tract cancer [16]. Blockade of PD-L1 with monoclonal antibody has shown promising results for increasing survival rates of patients with melanoma or renal cell cancer [17, 18].

Emerging evidence also suggested the prognostic role of PD-L1 expression in HNC patients. Several cohort studies showed that positive expression of PD-L1 was associated with poor survival of HNC patients [19–21]. However, other studies reached inconsistent conclusions. Budczies et al. [22] and Ock et al. [23] found no significant correlation between PD-L1 expression and HNC survival. Thierauf et al. [24] reported that HNC patients with PD-L1-positive expression even had a significantly longer survival. As controversies still remain, there is an urgent need to conduct a meta-analysis to clarify the prognostic value of PD-L1 in HNC patients and draw a firm conclusion to guide clinical practice.

## Materials and methods

### Literature search

The preferred reporting items for systematic reviews and meta-analyses (PRISMA) statement [25] and the reporting recommendations for tumor marker prognostic studies (REMARK) [26] were followed when we conducted and reported this meta-analysis. A comprehensive searching was carried out in electronic databases such as PubMed, EMBASE, Web of Science and the Cochrane Library from inception to August 2016. The search strategies were based on combinations of the following key words: “programmed cell death-ligand 1, PD-L1, CD274, B7-H1” AND “head and neck, oral, oropharyngeal, nasopharyngeal, laryngeal, pharynx” AND “tumor, neoplasm, cancer” AND “prognosis, survival, mortality”, without any restriction on language. Furthermore, we also used the corresponding Mesh terms such as “Antigens, CD274”, “Head and Neck Neoplasms” and “Survival”, “Prognosis”. The full electronic search strategy for PubMed database is detailed in S1 File. To identify additional potentially eligible studies, we also screened the reference lists of reviews and included articles.

### Selection of studies

Studies were considered eligible if they met the following criteria: (1) cohort studies on human beings; (2) investigated the correlation between PD-L1 expression and the survival of HNC patients; (3) reported or had sufficient information to estimate the hazard ratio (HR) and its 95% confidential interval (CI) for overall survival (OS) or disease-free survival (DFS). Studies reported in reviews, letters, or conference abstracts were excluded. For the studies with duplicate data, only the most complete study was included in the analysis. Two reviewers (Ji Li and Youliang Xu) independently screened the titles and abstracts of literatures identified by the search strategy to exclude irrelevant publications. The full texts of potentially eligible studies were then carefully examined to determine whether they were included or excluded. Disagreements were resolved by discussion with a third reviewer.

### Data extraction and quality assessment

Two reviewers (Ji Li and Ping Wang) independently extracted the following information using predefined data abstraction forms: first author, publication year, origin of population, subtype of HNC, type of PD-L1 expression, cut-off value for PD-L1 positive and patient survival data. For studies that showed survival data indirectly with a Kaplan-Meier curve, we used the methods described by Tierney et al. [27] to estimate.

The methodological quality of included studies was evaluated by two reviewers (Ji Li and Ping Wang) using the Newcastle-Ottawa quality assessment scale (NOS) for cohort study [28]. The scale included eight items of methodology, which were grouped into three categories: selection, comparability, and outcome. The final score (0–9) was assigned to each study based on these items. Any disagreements on the NOS score of the studies were resolved by discussion with a third reviewer.

### Statistical analysis

HRs with 95% CIs were used to assess the association between PD-L1 expression and the survival (including OS and DFS) of HNC patients. Heterogeneity among studies was assessed using *χ*^*2*^ test and the *I*^*2*^ statistic. We used a random-effects model to pool data if significant heterogeneity was detected (P<0.1 or *I*^*2*^>50%). Otherwise, we used a fixed-effects model. Subgroup analyses were carried out by subtype of HNC, type of PD-L1 expression, origin of population, and cut-off value for PD-L1 positive. Sensitive analyses were performed based on sample size, methods of HR estimation and calculation. The potential for publication bias was assessed by the Begg’s funnel plots and Egger’s regression test. All statistical analyses were performed using STATA 11.0 software (Stata Corporation, College Station, TX, USA), and a *P*-value less than 0.05 was considered as significant.

## Results

### Study selection

**Fig 1:**
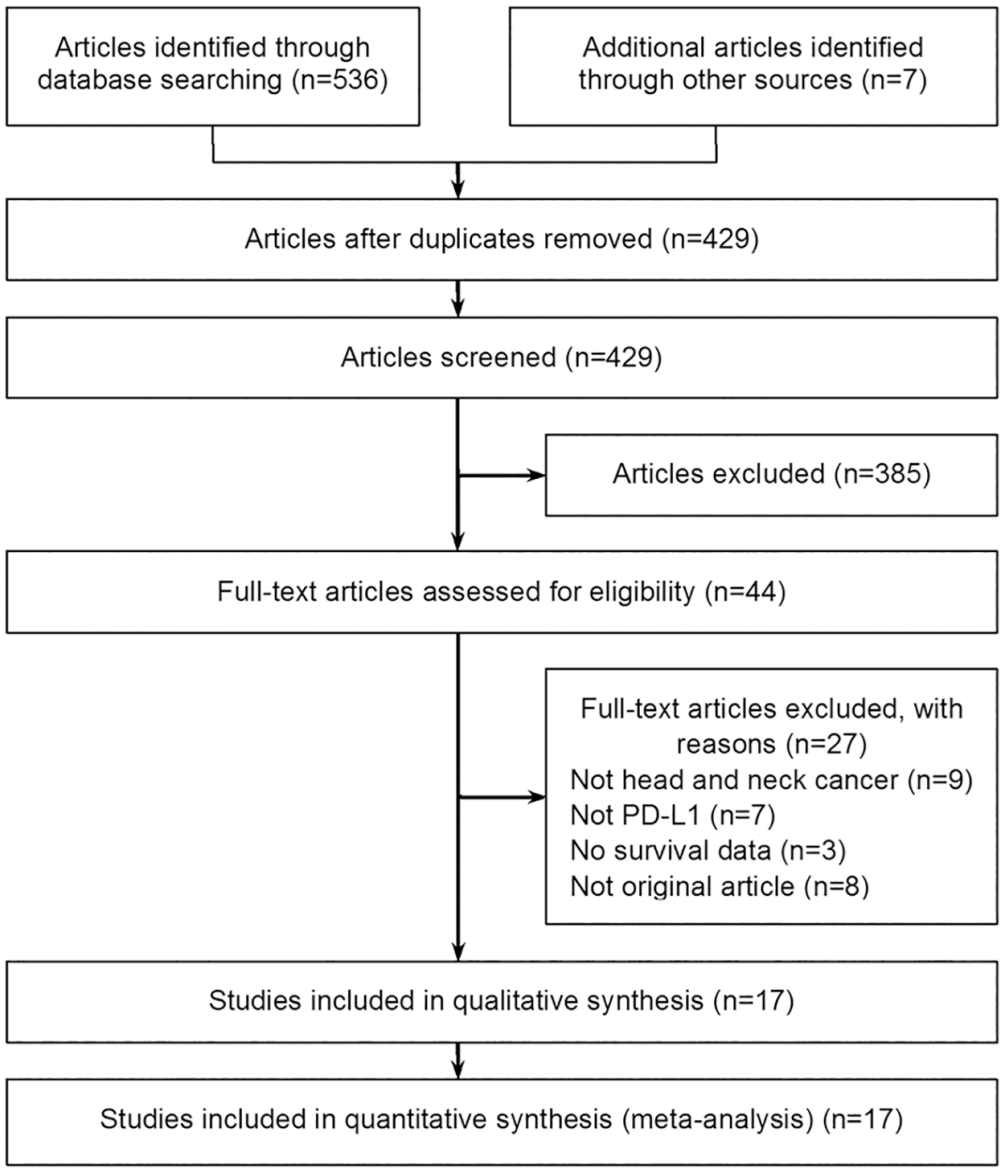
Flow chart of study selection. Comprehensive searching and selection of relevant articles.

A total of 536 articles were retrieved after searching the above databases, and additional searching identified seven articles. After removing the duplicates, 429 titles and abstracts were screened, and 385 records were excluded. Then we carefully read the full text of the remaining citations. 17 studies [5, 19–24, 29–38] investigating the correlation between PD-L1 expression and the survival of HNC patients were included. Fig 1 details the selection process.

### Characteristics and methodological quality of the included studies

Seventeen studies were included in the meta-analysis, among which nine studies [19, 21, 23, 29–34] investigated HNC patients from Asian countries/regions (including China, Japan, Korea, Taiwan and Hong Kong), five studies [5, 20, 22, 24, 38] investigated patients from European countries (including Germany, France and Greece), and three studies [35–37] investigated patients from other countries (USA, Australia and Brazil). The studies were published between 2011 and 2016, and the number of HNC patients they included ranged from 23 to 517. One study examined the gene expression of PD-L1 [22], while the others examined the protein expression [5, 19–21, 23, 24, 29–38]. Four studies included patients diagnosed with head and neck squamous cell carcinoma (HNSCC) [5, 22–24] while the remaining studies included patients diagnosed with the subtypes of HNC: six studies included patients with oral squamous cell carcinoma (OSCC) [19, 20, 29, 33, 35, 36], three studies included patients with nasopharyngeal carcinoma (NPC) [21, 30, 32], two studies included patients with oropharyngeal squamous cell carcinoma (OPSCC) [31, 37], one study included patients with laryngeal squamous cell carcinoma (LSCC) [38], and one study included patients with salivary gland carcinoma (SGC) [34]. Fourteen studies [5, 19, 20, 22, 23, 29, 31–38] showed the data of OS, and 11 studies [19–22, 24, 30–32, 34, 36, 38] showed the data of DFS. HRs with 95% CIs were obtained from the reports in ten studies [5, 19, 22, 23, 31–35, 38], and calculated from available data in seven studies [20, 21, 24, 29, 30, 36, 37]. Fourteen studies [5, 20–24, 29–33, 35–37] provided the survival data calculated by univariate analysis, two studies [19, 34] provided the data calculated by multivariate analysis, and one study [38] provided both the data calculated by univariate analysis and by multivariate analysis.

The methodological quality of included studies was rated by NOS score. The total scores they achieved ranged from 6 to 9. The median scores were 8 (interquartile range [IQR], 8 to 8). The characteristics and methodological quality of included studies are shown in Table 1.

**Table 1:** Main characteristics of studies included in the meta-analysis.

Author	Year	Origin of population	Subtype of HNC	No. of patients	PD-L1 expression	Cut-off value for PD-L1 positive	No. of positive expression	Survival parameter	HR estimation	HR calculation	NOS score
Badoual	2013	France	HNSCC	64	Protein	Score > 1	33	OS	Reported	Univariate analysis	8
Budczies	2016	Germany	HNSCC	517	mRNA	> median	220	OS, DFS	Reported	Univariate analysis	6
Chen	2015	Taiwan	OSCC	218	Protein	> 5%	139	OS, DFS	Reported	Multivariate analysis	8
Cho	2011	Korea	OSCC	45	Protein	Score > 2	26	OS	Calculated	Univariate analysis	8
Fang	2014	China	NPC	139	Protein	H score > 35	62	DFS	Calculated	Univariate analysis	8
Kim	2016	Korea	OPSCC	133	Protein	> 5%, > 20%	90	OS, DFS	Reported	Univariate analysis	9
Lee	2016	Hong Kong	NPC	104	Protein	> 25%	22	OS, DFS	Reported	Univariate analysis	8
Lin	2015	Taiwan	OSCC	305	Protein	Score > 1	133	OS	Reported	Univariate analysis	8
Mukaigawa	2016	Japan	SGC	219	Protein	> 1%	50	OS, DFS	Reported	Multivariate analysis	8
Ock	2016	Korea	HNSCC	141	Protein	> 5%	91	OS	Reported	Univariate analysis	8
Oliveira-Costa	2015	Brazil	OSCC	96	Protein	> 5%	47	OS	Reported	Univariate analysis	8
Satgunaseelan	2016	Australia	OSCC	217	Protein	> 5%	40	OS, DFS	Calculated	Univariate analysis	8
Straub	2016	Germany[22]	OSCC	90	Protein	> 5%	36	OS, DFS	Calculated	Univariate analysis	7
Thierauf	2015	Germany	HNSCC	23	Protein	> 10%	3	DFS	Calculated	Univariate analysis	6
Ukpo	2013	USA	OPSCC	181	Protein	> 5%	84	OS	Calculated	Univariate analysis	7
Vassilakopoulou	2016	Greece	LSCC	238	Protein	> 59th percentile of AQUA score	100	OS, DFS	Reported	Univariate and multivariate analysis	8
Zhang	2015	China	NPC	139	Protein	H score > 35	58	DFS	Calculated	Univariate analysis	8

HNC, head and neck cancer; HNSCC, head and neck squamous cell carcinoma; OSCC, oral squamous cell carcinoma; NPC, nasopharyngeal carcinoma; OPSCC, oropharyngeal squamous cell carcinoma; SGC, salivary gland carcinoma; LSCC, laryngeal squamous cell carcinoma; PD-L1, programmed cell death ligand 1; AQUA, automated quantitative protein analysis; OS, overall survival; DFS, disease-free survival; HR, hazard ratio; NOS, the Newcastle-Ottawa Scale.

### Association between PD-L1 expression and OS of HNC patients

**Fig 2:**
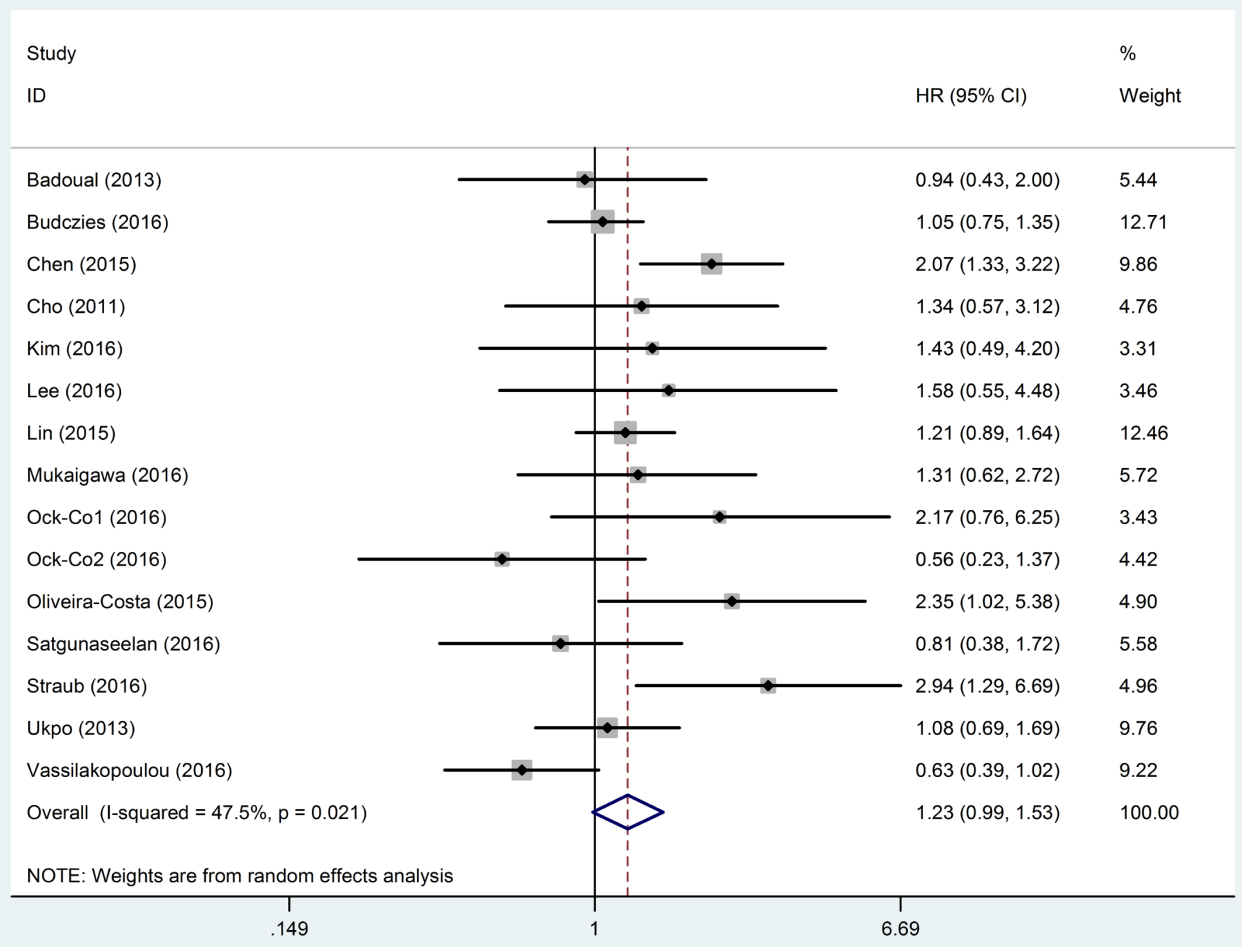
Meta-analysis of programmed cell death ligand 1 (PD-L1) expression and the overall survival (OS) of head and neck cancer (HNC) patients. Significant heterogeneity existed among the studies reporting the outcome of OS (*I*^*2*^ = 47.5%, *P* = 0.021). Therefore, a random-effects model was applied to pool the data and the results showed that there was no significant correlation between PD-L1 expression and OS of HNC patients (HR, 1.23; 95% CI, 0.99–1.53). The horizontal bars indicate the 95% CIs. The size of the square indicates the weight of the individual study in the meta-analysis. The blue hollow diamond indicates the pooled HR (the center of the diamond) with 95% CI (the extremities of the diamond). HR, hazard ratio; CI: confidence interval.

HRs with 95% CIs of OS obtained from 14 studies [5, 19, 20, 22, 23, 29, 31–38] were pooled to investigate the association between PD-L1 expression and OS of HNC patients. As significant heterogeneity was detected (*I*^*2*^ = 47.5%, *P* = 0.021) among studies, the data were pooled with a random-effects model being used. The results showed that there was no significant difference between positive and negative expression of PD-L1 on the OS of HNC patients (HR, 1.23; 95% CI, 0.99–1.53; *P* = 0.065) (Fig 2). Subgroup analysis by subtype of HNC was performed. The results showed significant association between PD-L1 expression and OS in patients diagnosed with OSCC, while for patients with the other types of HNC (i.e. HNSCC, OPSCC, LSCC, NPC, and SGC), no significant association was observed. Positive expression of PD-L1 was also associated with poor OS in HNC patients from Asian countries/regions, but had no association with OS in patients from European countries. Subgroup analyses were also carried out by type of PD-L1 expression and cut-off value for PD-L1 positive. However, the results showed that there was no significant correlation between PD-L1 expression and OS of HNC patients in all the subgroups. Sensitive analyses were performed based on sample size, methods of HR estimation and HR calculation. The results showed that the meta-analysis results were not altered by these factors. The results of subgroup analysis and sensitive analysis on the outcome of OS are shown in Table 2.

**Table 2:** Subgroup analysis and sensitive analysis on the outcome of overall survival.

	No. of Studies	No. of patients	Heterogeneity	Statistical model used	HR (95% CI)	Significance
**Subtype of HNC**						
1. HNSCC	3	722	*I*^*2*^ = 20.8%; *P* = 0.285	Fixed	1.03 (0.80, 1.33)	*P* = 0.827
2. OSCC	6	971	*I*^*2*^ = 51.4%; *P* = 0.067	Random	1.58 (1.11, 2.25)	*P* = 0.011
3. OPSCC	2	314	*I*^*2*^ = 0.0%; *P* = 0.637	Fixed	1.13 (0.75, 1.71)	*P* = 0.568
4. LSCC	1	238	NA	Fixed	0.63 (0.39, 1.03)	*P* = 0.063
5. NPC	1	104	NA	Fixed	1.58 (0.56, 4.51)	*P* = 0.390
6. SGC	1	219	NA	Fixed	1.31 (0.63, 2.74)	*P* = 0.474
**PD-L1 expression**						
1. mRNA	1	517	NA	Fixed	1.05 (0.75, 1.35)	*P* = 0.745
2. Protein	13	2051	*I*^*2*^ = 49.5%; *P* = 0.018	Random	1.26 (0.98, 1.62)	*P* = 0.069
**Origin of population**						
1. Europe	4	909	*I*^*2*^ = 70.6%; *P* = 0.017	Random	1.08 (0.66, 1.78)	*P* = 0.758
2. Asia	7	1165	*I*^*2*^ = 19.2%; *P* = 0.278	Fixed	1.38 (1.12, 1.70)	*P* = 0.003
**Cut-off value for PD-L1 positive**						
1. > 5%	7	1076	*I*^*2*^ = 54.8%; *P* = 0.030	Random	1.46 (1.00, 2.14)	*P* = 0.051
2. Score > 1	2	369	*I*^*2*^ = 0.0%; *P* = 0.551	Fixed	1.17 (0.88, 1.55)	*P* = 0.285
**Sample size of studies**						
1. < 100	5	436	*I*^*2*^ = 51.6%; *P* = 0.067	Random	1.47 (0.89, 2.44)	*P* = 0.134
2. > 100	9	2132	*I*^*2*^ = 45.6%; *P* = 0.065	Random	1.15 (0.91, 1.44)	*P* = 0.251
**HR estimation**						
1. Reported	10	2035	*I*^*2*^ = 51.7%; *P* = 0.023	Random	1.21 (0.93, 1.57)	*P* = 0.148
2. Available data calculated	4	533	*I*^*2*^ = 49.0%; *P* = 0.118	Random	1.30 (0.80, 2.12)	*P* = 0.289
**HR Calculation**						
1. Multivariate analysis	3	675	*I*^*2*^ = 84.9%; *P* = 0.001	Random	1.15 (0.50, 2.67)	*P* = 0.725
2. Univariate analysis	12	2131	*I*^*2*^ = 39.2%; *P* = 0.072	Random	1.14 (0.91, 1.43)	*P* = 0.241

HNC, head and neck cancer; HNSCC, head and neck squamous cell carcinoma; OSCC, oral squamous cell carcinoma; OPSCC, oropharyngeal squamous cell carcinoma; LSCC, laryngeal squamous cell carcinoma; NPC, nasopharyngeal carcinoma; SGC, salivary gland carcinoma; PD-L1, programmed cell death ligand 1; HR, hazard ratio; CI, confidence interval; NA, not applicable.

### Association between PD-L1 expression and DFS of HNC patients

**Fig 3:**
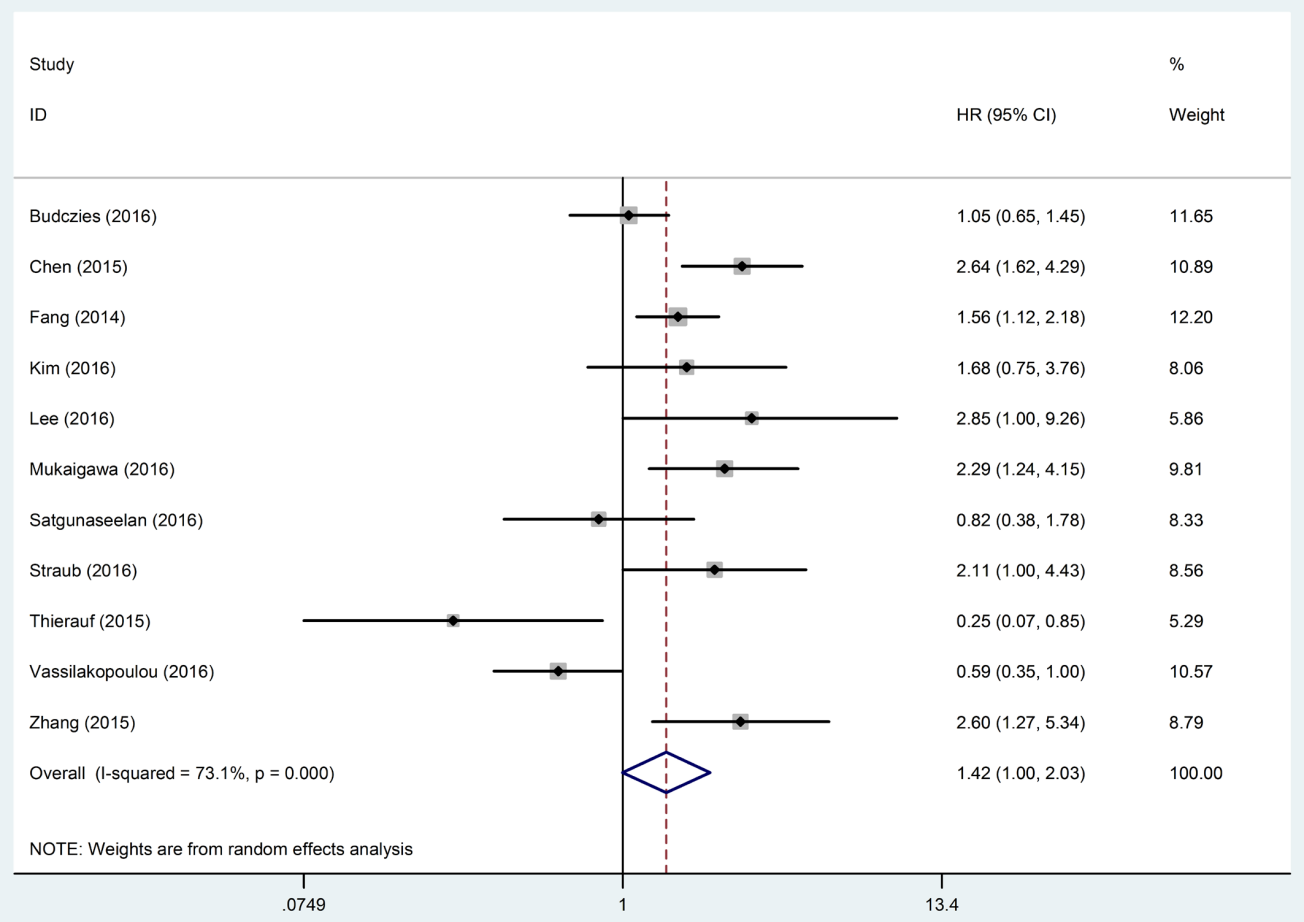
Meta-analysis of programmed cell death ligand 1 (PD-L1) expression and the disease-free survival (DFS) of head and neck cancer (HNC) patients. Significant heterogeneity existed among the studies reporting the outcome of DFS (*I*^*2*^ = 73.1%, *P* < 0.001). Therefore, a random-effects model was applied to pool the data and the results showed that there was no significant correlation between PD-L1 expression and DFS of HNC patients (HR, 1.42; 95% CI, 1.00–2.03). The horizontal bars indicate the 95% CIs. The size of the square indicates the weight of the individual study in the meta-analysis. The blue hollow diamond indicates the pooled HR (the center of the diamond) with 95% CI (the extremities of the diamond). HR, hazard ratio; CI: confidence interval.

Eleven studies [19–22, 24, 30–32, 34, 36, 38] provided the data of DFS, but significant heterogeneity existed among these studies (*I*^*2*^ = 73.1%, *P* < 0.001). Therefore, a random-effects model was applied to pool the data. The results showed that there was no significant correlation between PD-L1 expression and DFS of HNC patients (HR, 1.42; 95% CI, 1.00–2.03; *P* = 0.052) (Fig 3). The results of subgroup analysis by subtype of HNC showed that positive expression of PD-L1 was associated with poor DFS in patients diagnosed with LSCC, NPC and SGC, but had no association with DFS in patients diagnosed with HNSCC, OSCC, or OPSCC. Significant association between PD-L1 and DFS was observed in HNC patients from Asian countries/regions, but not in patients from European countries. Subgroup analysis by cut-off value for PD-L1 was conducted. The pooled result of the studies defining PD-L1 positive as H score>35 suggested a significant correlation between PD-L1 expression and DFS of HNC patients. Subgroup analyses were also performed by type of PD-L1 expression. However, the results showed that there was no significant correlation between PD-L1 expression (both gene and protein) and OS of HNC patients. When performing sensitive analysis based on sample size, the combined HR was only statistically significant in large-sample size (n > 100) studies. As for the methods of HR calculation, the HRs that were calculated by multivariate survival analysis were pooled, and the results showed that positive expression of PD-L1 was associated with poor DFS in HNC patients. Sensitive analyses were also performed by method of HR estimation, but the results showed that the meta-analysis results were not altered by this factor. The results of subgroup analysis and sensitive analysis on the outcome of DFS are shown in Table 3.

**Table 3:** Subgroup analysis and sensitive analysis on the outcome of disease-free survival.

	No. of Studies	No. of patients	Heterogeneity	Statistical model used	HR (95% CI)	Significance
**Subtype of HNC**						
1. HNSCC	2	540	*I*^*2*^ = 79.1%; *P* = 0.028	Random	0.58 (0.15, 2.30)	*P* = 0.438
2. OSCC	3	525	*I*^*2*^ = 68.5%; *P* = 0.042	Random	1.73 (0.88, 3.42)	*P* = 0.112
3. OPSCC	1	133	NA	Fixed	1.68 (0.75, 3.76)	*P* = 0.206
4. LSCC	1	238	NA	Fixed	0.59 (0.35, 1.00)	*P* = 0.048
5. NPC	3	382	*I*^*2*^ = 15.0%; *P* = 0.308	Fixed	1.77 (1.32, 2.37)	*P* = 0.001
6. SGC	1	219	NA	Fixed	2.29 (1.25, 4.19)	*P* = 0.007
**PD-L1 expression**						
1. mRNA	1	517	NA	Fixed	1.05 (0.65, 1.45)	*P* = 0.812
2. Protein	10	1520	*I*^*2*^ = 73.8%; *P* < 0.001	Random	1.48 (0.99, 2.20)	*P* = 0.056
**Origin of population**						
1. Europe	4	868	*I*^*2*^ = 75.6%; *P* = 0.006	Random	0.84 (0.44, 1.61)	*P* = 0.598
2. Asia	6	952	*I*^*2*^ = 0.0%; *P* = 0.463	Fixed	1.99 (1.59, 2.48)	*P* = 0.001
**Cut-off value for PD-L1 positive**						
1. > 5% positive expression	3	525	*I*^*2*^ = 68.5%; *P* = 0.042	Random	1.73 (0.88, 3.42)	*P* = 0.112
2. H score > 35	2	278	*I*^*2*^ = 37.6%; *P* = 0.206	Fixed	1.71 (1.26, 2.32)	*P* = 0.011
**Sample size of studies**						
1. < 100	2	113	*I*^*2*^ = 88.3%; *P* = 0.003	Random	0.77 (0.10, 6.15)	*P* = 0.806
2. > 100	9	1924	*I*^*2*^ = 71.7%; *P* < 0.001	Random	1.52 (1.06, 2.17)	*P* = 0.022
**HR estimation**						
1. Reported	6	1429	*I*^*2*^ = 78.6%; *P* < 0.001	Random	1.54 (0.91, 2.60)	*P* = 0.106
2. Available data calculated	5	608	*I*^*2*^ = 70.9%; *P* = 0.008	Random	1.29 (0.73, 2.28)	*P* = 0.386
**HR Calculation**						
1. Multivariate analysis	2	437	*I*^*2*^ = 0.0%; *P* = 0.719	Fixed	2.50 (1.71, 3.65)	*P* = 0.001
2. Univariate analysis	9	1600	*I*^*2*^ = 70.0%; *P* = 0.001	Random	1.23 (0.84, 1.82)	*P* = 0.289

HNC, head and neck cancer; HNSCC, head and neck squamous cell carcinoma; OSCC, oral squamous cell carcinoma; OPSCC, oropharyngeal squamous cell carcinoma; LSCC, laryngeal squamous cell carcinoma; NPC, nasopharyngeal carcinoma; SGC, salivary gland carcinoma; PD-L1, programmed cell death ligand 1; HR, hazard ratio; CI, confidence interval; NA, not applicable.

### Publication bias

**Fig 4:**
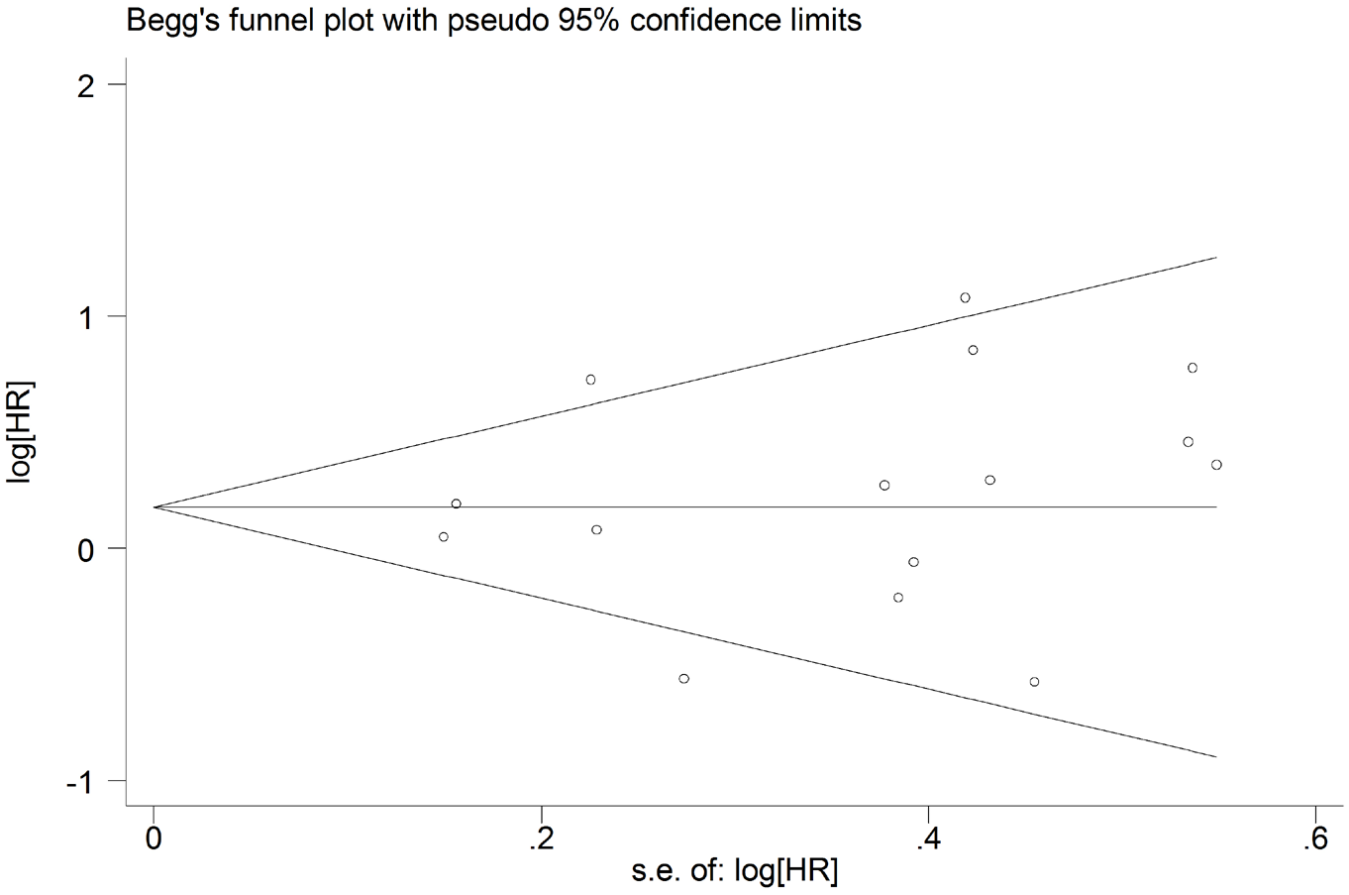
Begg’s funnel plot for assessment of potential publication bias in studies investigating the correlation between programmed cell death ligand 1 (PD-L1) expression and the overall survival of head and neck cancer patients. No evidence of publication bias was observed, as indicated by a symmetric funnel plot (Begg’s *P* = 0.373). HR, hazard ratio.

**Fig 5:**
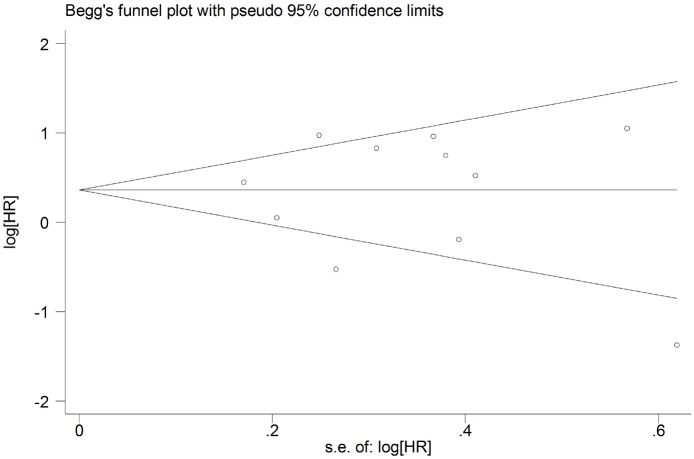
Begg’s funnel plot for assessment of potential publication bias in studies investigating the correlation between programmed cell death ligand 1 (PD-L1) expression and the disease-free survival of head and neck cancer patients. No evidence of publication bias was observed, as indicated by a symmetric funnel plot (Begg’s *P* = 0.640). HR, hazard ratio.

**Fig 6:**
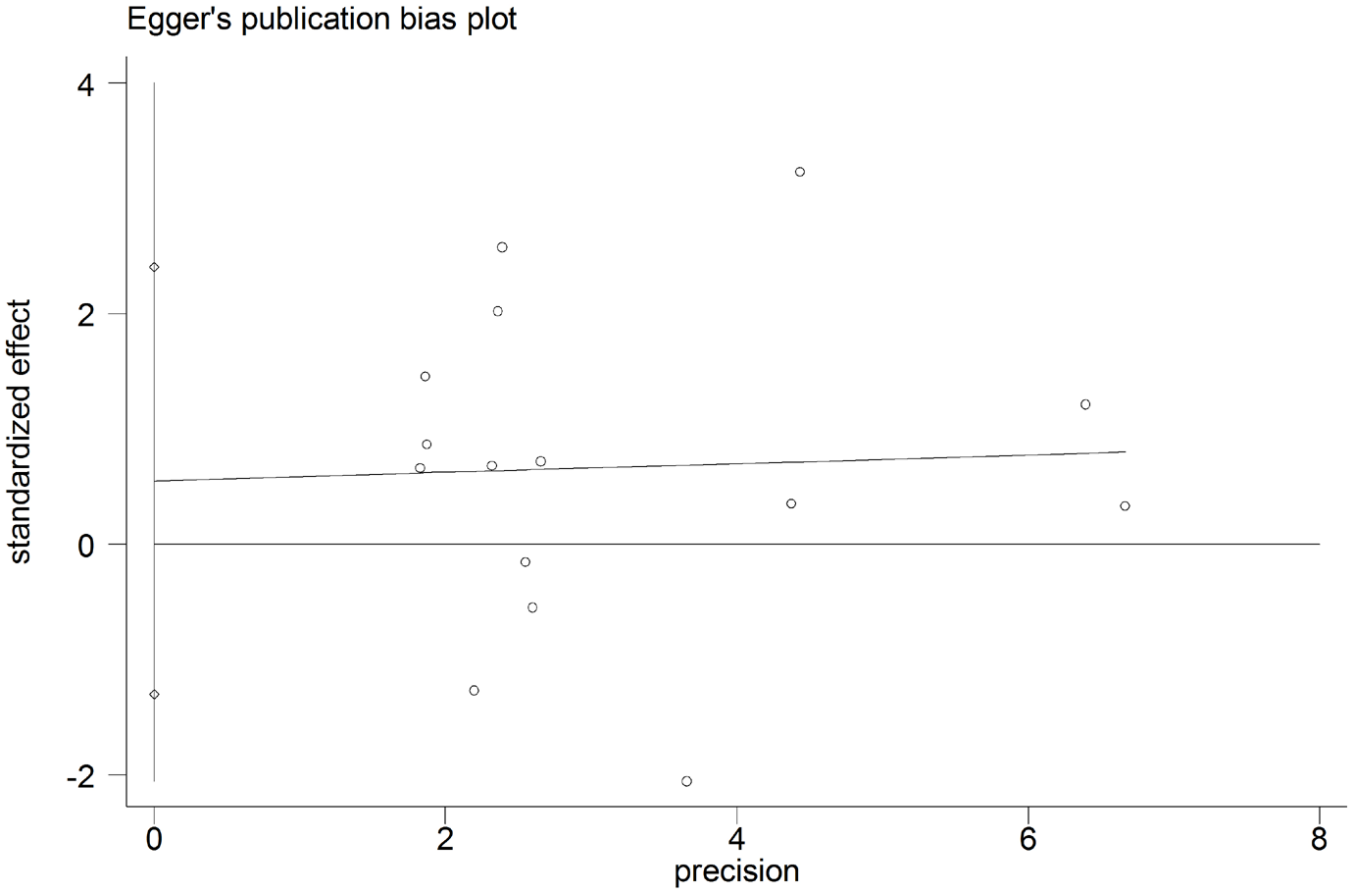
Egger’s test for assessment of potential publication bias in studies investigating the correlation between programmed cell death ligand 1 (PD-L1) expression and the overall survival of head and neck cancer patients. Egger's test revealed no evidence of publication bias (Egger’s *P* = 0.880) among the studies reporting the outcome of overall survival.

**Fig 7:**
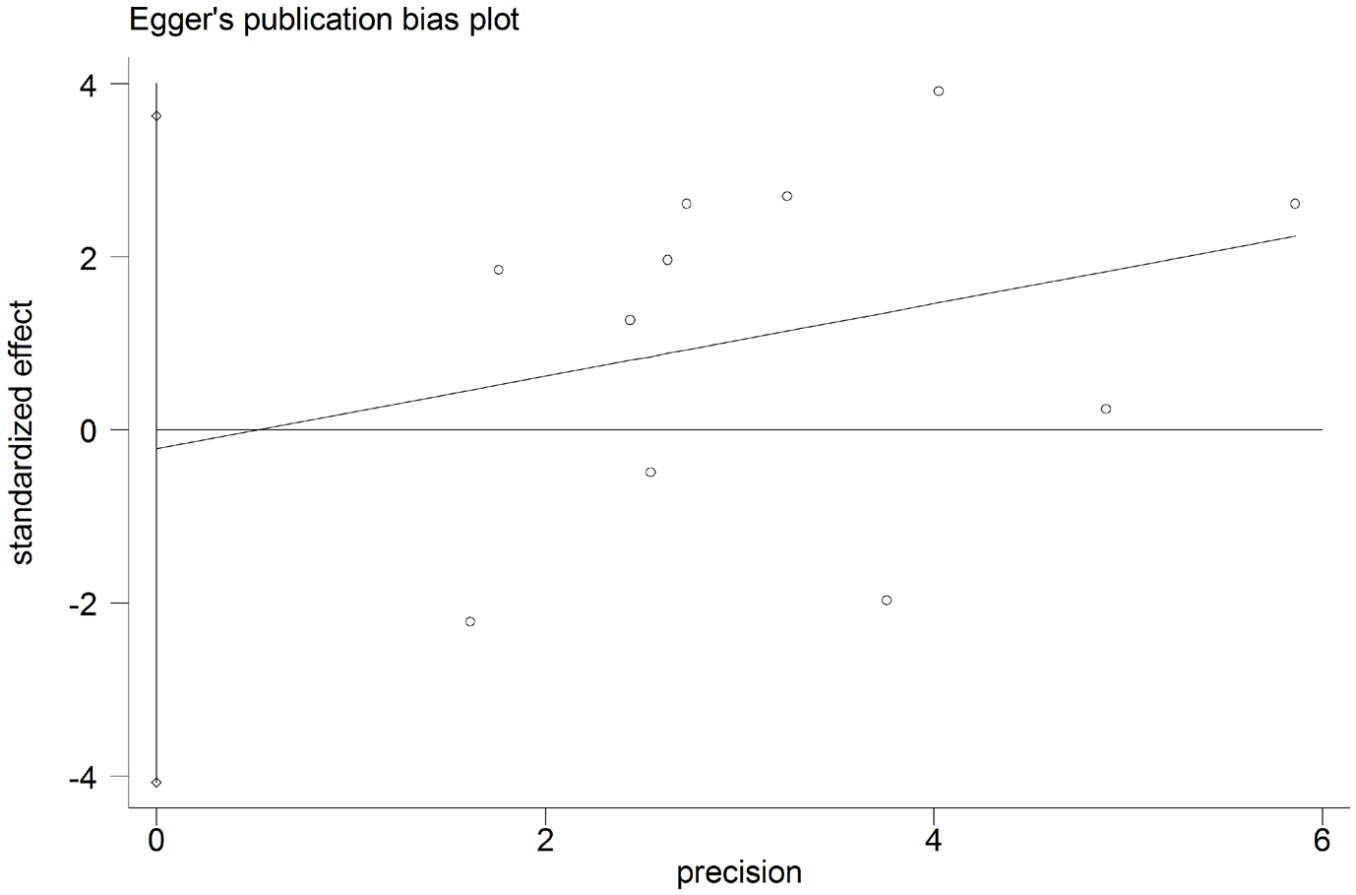
Egger’s test for assessment of potential publication bias in studies investigating the correlation between programmed cell death ligand 1 (PD-L1) expression and the disease-free survival of head and neck cancer patients. Egger's test revealed no evidence of publication bias (Egger’s *P* = 0.416) among the studies reporting the outcome of disease-free survival.

The potential for publication bias was assessed by the Begg’s funnel plots and Egger’s regression test. As shown in Figs 4 and 5, funnel plot asymmetry was not found on the outcomes of OS (Begg’s *P* = 0.373) and DFS (Begg’s *P* = 0.640). The Egger’s tests also failed to reveal the significant evidence of publication bias in OS (Egger’s *P* = 0.880) (Fig 6) and DFS (Egger’s *P* = 0.416) (Fig 7).

## Discussion

PD-L1 expression was reported to be correlated with poor prognosis in various cancers [14–16, 39]. However, the relationship between PD-L1 expression and the prognosis of patients with HNC remains inconclusive. In the present study, we aimed to clarify the prognostic value of PD-L1 in HNC patients using meta-analysis techniques. To our knowledge, it is the first meta-analysis to systematically analyze the correlation between PD-L1 expression and the survival of HNC patients. The study analyzed the survival data of 2,869 HNC patients from 17 cohort studies [5, 19–24, 29–38], and the results showed that there was no significant difference between positive and negative expression of PD-L1 on either the OS or the DFS of HNC patients. However, the subgroup analysis suggested that positive expression of PD-L1 was associated with poor OS and DFS in HNC patients from Asian countries/regions. When performing subgroup analysis by subtype of HNC, we found that positive expression of PD-L1 correlated with poor OS in patients with OSCC, and also correlated with poor DFS in patients diagnosed with LSCC, NPC and SGC.

PD-L1 is an important immune regulatory molecule that was recently reported to be critically implicated in the immune escape mechanism of cancer cells [9]. The lack of immunologic control is currently recognized as a distinct hallmark of cancer [6], and the process of cancer immunoediting, which involves phases of elimination, equilibrium and escape, has been proposed as a mechanism by which tumors escape control [40]. Under normal circumstance, the host immune homeostasis was maintained by the balance between co-stimulatory and co-inhibitory signals. PD-1/PD-L1 is an important co-inhibitory signaling pathway aiming to protect healthy cells from excessive inflammatory or autoimmune responses [11, 12]. However, this signaling pathway was also reported to be involved in the equilibrium and escape phases of cancer immunoediting to promote the survival and growth of tumor cells [41]. PD-1 is a member of the B7/CD28 co-stimulatory factor superfamily that is widely expressed on immune cells such as T cells, B cells and NK cells. PD-L1 (also known as CD274 or B7 homolog 1) is a ligand of PD-1. It is expressed not only on activated T cells, B cells, dendritic cells, macrophages, but also on various tumor cells. Interactions between PD-L1 and PD-1 were reported to protect the tumor cells through the following mechanisms [14]. First, PD-L1 could ligate PD-1 on antigen specific T cells and leads to functional anergy or death of these effector T cells [42]. Second, interactions between PD-L1 and PD-1 could directly protect the tumor cells from apoptosis by reverse signaling through PD-L1 [43, 44]. Third, by ligation of PD-1 on regulatory T cells, PD-L1 could inhibits their ability to mediate tolerance [13]. Fourth, PD-L1 could also inhibit the immune response of cytotoxic T cells by interactions with CD80 [45]. The above evidence demonstrated that PD-L1 expression was implicated in the pathogenesis of cancers, and also suggested that positive expression of PD-L1 may be associated with poor prognosis of cancers.

Actually, the association between the expression of PD-L1 and survival in patients with cancers has been widely investigated. Recently, several studies systematically reviewed the current available evidence. In the meta-analysis conducted by Li et al. [14], they analyzed the survival data of 7,802 patients with breast cancer, and found that PD-L1 protein expression was associated with shorter OS, shorter DFS, and shorter metastasis-free survival in these patients. They concluded that high PD-L1 protein expression appeared to be a negative prognostic factor in breast cancer. Similar results were observed in patients with gastrointestinal tract cancer. Huang et al. [16] pooled the data from fifteen studies that involved 2,993 gastrointestinal tract cancer patients. The results indicated that positive PD-L1 expression status in tumor cells was a risk factor for prognosis in gastrointestinal tract cancer, especially in esophageal cancer. Besides, the prognostic value of PD-L1 expression in other tumors, such as gastric cancer [39] and renal cell carcinoma [15] were also demonstrated by meta-analysis. However, Wu et al. [46] found that the correlations between PD-L1 and prognosis are variant among different tumor types. The results of meta-analysis conducted by Zhong et al. [47] showed that PD-L1 expression did not correlate with prognosis in terms of OS in patients with non-small-cell lung cancer.

In the present study, we found that there was no significant correlation between PD-L1 expression and OS or DFS of HNC patients. However, the subgroup analysis showed that positive expression of PD-L1 was associated with poor survival in HNC patients from Asian countries/regions. Subgroup analysis by subtype of HNC showed that positive expression of PD-L1 may correlate with poor OS in patients with OSCC, and correlate with poor DFS in patients diagnosed with LSCC, NPC and SGC. The results suggested that positive expression of PD-L1 may predict poor prognosis in these subpopulations of HNC patients. Moreover, these findings would also help to establish the rationale on the usage of immunotherapies targeting the PD-1/PD-L1 pathway for these patients [48]. A recent study assessed the tolerability and antitumour efficacy of a humanized PD-1 antibody, pembrolizumab, in HNC [49]. By analyzing the data of 104 patients, the authors concluded that pembrolizumab was well tolerated and demonstrated clinically meaningful antitumour activity in patients with PD-L1-positive recurrent or metastatic HNSCC. The study suggested the therapeutic potential of targeting the PD-1/PD-L1 immune checkpoint in the subpopulations of HNC patients. Therefore, the findings of our study may have clinical implications to guide the optimal clinical application of PD-1/PD-L1 inhibitor in HNC patients.

To test the robustness of the results of meta-analysis, we conducted sensitive analysis on the outcomes of OS and DFS by sample size (large-sample vs small-sample), methods of HR estimation (directly reported vs calculated) and HR calculation (multivariate analysis vs univariate analysis). However, the sensitive analyses suggested that the results of meta-analyses were not robust, so we failed to draw a firm conclusion on the relationship between PD-L1 expression and the prognosis of HNC patients. This is a limitation of our study. Moreover, other two limitations may also exist in the present study. First, only eight [5, 21, 29–31, 33, 34, 38] of the included studies reported that the PD-L1 expression was blindly observed by investigators, which may introduce detection bias to the meta-analysis. Second, the cut-off values for PD-L1 positivity varied among the included studies, so we failed to determine the optimal cut-off value for it. Future studies are suggested to choose a well-recognized cut-off value to define the positive expression of PD-L1.

## Conclusions

Our meta-analysis indicated that positive expression of PD-L1 could serve as a good predictor for poor prognosis of Asian patients with HNC. However, the findings still need to be confirmed by large-scale, prospective studies. Future studies also need to determine the optimal cut-off value to define the positive expression of PD-L1.

## Supporting information

S1 File Full electronic search strategy for PubMed database. (DOCX)

S1 Table PRISMA 2009 checklist for this meta-analysis. (DOC)
