# Expression of Concern: Prognostic value of programmed cell death ligand 1 expression in patients with head and neck cancer: A systematic review and meta-analysis

**DOI:** 10.1371/journal.pone.0278502

**Published:** 2022-11-28

**Authors:** The PLOS ONE Editors

After this article was published, similarities were noted between this article [1] and submissions by other research groups which call into question the validity and provenance of the reported results. In light of these issues, the *PLOS ONE* Editors issue this Expression of Concern.

PLOS attempted to discuss the concerns with the authors, but the authors either did not reply or could not be reached through the email addresses on file with the journal.
