# Supplementary material for: Prognostic value of programmed cell death ligand 1 expression in patients with head and neck cancer: A systematic review and meta-analysis
Source: PLoS One. 2017 Jun 12;12(6):e0179536. doi: 10.1371/journal.pone.0179536 (PMC5467853; doi:10.1371/journal.pone.0179536)
Supplement: S1 File — (DOCX) [file pone.0179536.s001.docx]

Full electronic search strategy for PubMed database

#1 PD-L1[Title/Abstract]) OR CD274[Title/Abstract]) OR B7-H1[Title/Abstract]) OR B7 homolog 1[Title/Abstract]) OR programmed death-ligand 1[Title/Abstract]) OR programmed cell death-ligand 1[Title/Abstract]

#2 "Antigens, CD274"[Mesh] OR "PDCD1 protein, human" [Supplementary Concept] OR "CD274 protein, human" [Supplementary Concept] OR "Programmed Cell Death 1 Receptor"[Mesh]

#3 #1 OR #2

#4 oral[Title/Abstract]) OR lip[Title/Abstract]) OR pharynx[Title/Abstract]) OR nasopharyngeal[Title/Abstract]) OR salivary gland[Title/Abstract]) OR pharyngeal[Title/Abstract]) OR laryngeal[Title/Abstract]) OR larynx[Title/Abstract]) OR nasopharynx[Title/Abstract]) OR oralpharynx[Title/Abstract]) OR hypopharynx[Title/Abstract]) OR sinonasal[Title/Abstract]) OR tongue[Title/Abstract]) OR oropharynx[Title/Abstract]) OR buccal[Title/Abstract]) OR mouth[Title/Abstract]) OR (head and neck[Title/Abstract])

#5 cancer[Title/Abstract]) OR cancers[Title/Abstract]) OR neoplasm[Title/Abstract]) OR carcinoma[Title/Abstract]) OR neoplasms[Title/Abstract]) OR tumor[Title/Abstract]) OR carcinomas[Title/Abstract]) OR tumors[Title/Abstract]

#6 #4 AND #5

#7 "Head and Neck Neoplasms"[Mesh]

#8 #6 OR #7

#9 prognos*[Text Word] OR mortality[Text Word] OR surviv*[Text Word] OR hazard[Text Word] OR predict*[Text Word]

#10 "Survival"[Mesh] OR "Mortality"[Mesh] OR "Survival Analysis"[Mesh] OR "Disease-Free Survival"[Mesh] OR "Prognosis"[Mesh]

#11 #9 OR #10

#12 #3 AND #8 AND #11
